# Stop Turning a Blind Eye: Tobacco Smoking Among Egyptian Patients With Schizophrenia

**DOI:** 10.3389/fpsyt.2018.00703

**Published:** 2019-01-04

**Authors:** Hussien Elkholy, Nahla Nagy, Ghada R. A. Taha, Mahmoud Elhabiby, Mostafa Yosef, Lobna Azzam

**Affiliations:** ^1^Neurology and Psychiatry Department, Faculty of Medicine, Ain Shams University, Cairo, Egypt; ^2^Community Medicine Department, Faculty of Medicine, Ain Shams University, Cairo, Egypt

**Keywords:** tobacco smoking, nicotine, schizophrenia, motivation to quit, mortality, positive symptoms, PANSS

## Abstract

**Background:** Patients with schizophrenia have considerably higher rates of mortality than general population. Multiple factors may play a role in this. Despite being a major preventable cause of death, smoking is usually overlooked when dealing with patients with schizophrenia. Understanding the pattern of smoking, its severity, and the reasons to quit might be helpful in managing patients with schizophrenia and decreasing the mortality gap.

**Subjects and Methods:** The study included smokers divided into two groups; the first included 346 patients with schizophrenia while the second group had 150 smokers with no mental illness. Both groups were assessed and compared regarding sociodemographic variables, pattern of smoking, severity of nicotine dependence, and motivation to quit smoking.

**Results:** Earlier age of starting to smoke, higher number of cigarettes per day, and lower dependency scores were noted in patients with Schizophrenia. Positive correlation was found between positive symptoms and severity of dependence. Specific positive symptoms were correlated to number of cigarettes per day and time before first cigarette. Patients with Schizophrenia showed a significant difference in intrinsic reasons to quit (health concerns and self-control), which were also positively correlated to their positive symptoms score. Linear regression analysis for predictors of FTND score revealed that only age, sex, and schizophrenia were significant predictors of FTND score.

**Conclusion:** Patients with schizophrenia smoke at earlier ages and smoke more cigarettes per day, yet, have less severe dependence than non-schizophrenic counterparts. Positive symptoms play a role in their smoking pattern and severity. Health concerns and self-control are their main motives to quit smoking.

## Introduction

Mortality gap in schizophrenia is a significant issue (1). Physical illness is associated with the problem, which may be related to the adoption of unhealthy lifestyle behaviors (2).

Smoking is one of the main factors adding to the mortality gap in patients with schizophrenia being one of the leading causes of premature mortality in them (3). Yet, it is usually overlooked. Tobacco smoking is common in schizophrenia (4). Patients with schizophrenia have high reported smoking rates of 58–90% (5). This is considerably higher than smoking rates of 24% in the general public (6) and of ~50% in people with other psychiatric diagnoses (7). Patients with schizophrenia are not only more likely to smoke, but also smoke more heavily, have longer smoking duration, and have lower rates of cessation compared to individuals without schizophrenia (8). Among different factors that may explain the high rates of smoking among patients with schizophrenia is the shortage of smoking cessation services in mental health treatment programs (9).

Smoking is a major preventable cause of death (10). In patients with schizophrenia, the risk of mortality is doubled, and the risk of cardiac-related morality is increased about 12-fold in smokers compared to non-smokers (1, 11). However, despite the high frequency of smoking in schizophrenia, little is known about clinical correlates or pharmacological treatments associated with tobacco smoking in patients with schizophrenia (12).

The relation between schizophrenia and smoking has been the subject of many studies for years. Some showed smoking to be associated with both positive and negative symptoms of schizophrenia, others found relationship with just one of these domains (13), while some found no relationship with either (14). Some authors suggest that Schizophrenia does not only increase the possibility of smoking but increases the risk of severe smoking as well (15).

Consequently, the high rates and severer patterns of smoking in patients with schizophrenia are suggested to contribute to reduction in life expectancy and to excess morbidity and mortality among them. Moreover, cigarette smoking has also been demonstrated to be associated with more frequent hospital readmissions, increased financial burden, and higher rate of attempted suicide (16). Addressing several factors like lack of resources, smoking, physical illness, and lifestyle modification may help decreasing the mortality gap in schizophrenia (1).

On the other hand, studies suggest that patients with schizophrenia are at least as motivated to quit as the general population but have lesser chance to quit attempts (17, 18). Moreover, several studies suggest that tobacco dependence treatment is effective in patients with mental illness (9, 19, 20), with some exploring the safety and efficacy of pharmacological treatment in this group (21, 22). Despite that, most psychiatric services do not address smoking cessation in patients with schizophrenia. This may be due to the lack of a consensus on certain recommendations to treat tobacco dependence in this patients group. Moreover, only modest results are achieved with the available regular approaches for smoking cessation (23). However, specific programs that promote healthy living, e.g., LIFESTYLE trial (2) are emerging recently. Those programs might play a key role in helping patients with Schizophrenia getting rid of harmful habits like smoking which might decrease mortality rates.

Therefore, as smoking appears to be one of the major factors contributing to the mortality gap in patients with schizophrenia, this study aims at shedding the light on smoking patterns among a sample of patients with Schizophrenia in Egypt and to highlight the patients' willingness to quit.

## Materials and Methods

This is a comparative cross-sectional study between two different groups; the first included smokers with schizophrenia and the second had smokers without mental illness. The sample was a convenient sample collected over a 1-year period. The sample size was also calculated prior to the study using PASS version 15.0, setting the power at 80% and the significance level (α- error) at 0.05, to make sure the study reaches the minimal sample size. Data from previous study (24) reported that the mean immediate reinforcement score for patients with schizophrenia was 2.0 ± 1.1 compared to 2.3 ± 0.9 for normal controls. Calculation according to these values produced a minimal sample size of 139 subjects per group, rounded to 150 in each.

The first group; included 346 patients with schizophrenia who were collected during the study period using a convenient sampling method. Patients fitting the inclusion criteria were approached in both inpatients and outpatients departments, at the Institute of Psychiatry, Faculty of Medicine, Ain Shams University, and those who consented were included. This group included both sexes, older than 18 at the time of the study, with the diagnosis of Schizophrenia according to DSM IV-TR criteria and had been smokers for at least 12 months. Presence of any other Axis I psychiatric diagnosis was an exclusion criterion.

As for group two; 150 subjects were recruited from the smoking cessation clinic and we also called for volunteers from employees and students through an announcement in the university hospitals. This group included both sexes, above the age of 18 with exclusion of those with any Axis I psychiatric disorder.

Required ethical approvals were obtained prior to the study. Informed written consents were requested from all participants, including detailed description of the study procedures. Socio-demographic data were collected using a questionnaire based on the admission sheet of the Institute of Psychiatry, Ain Shams University.

Further assessment was done using the following. (a) Structured Clinical Interview for DSM-IV Axis-I disorders (SCID) (25), an Arabic version validated in Egyptian studies was used (26) to confirm the diagnosis of Schizophrenia and to exclude other Axis I psychiatric disorders in both groups. (b) Positive and Negative Syndrome Scale (PANSS) (27). The Positive and Negative Syndrome Scale is one of the most widely used methods for standardized measurement of schizophrenic core symptoms. The PANSS consists of 7 positive symptom items, 7 negative symptom items, and 16 general psychopathology items. All 30 PANSS items are rated on a 7-point symptom severity scale, ranking from 1 (absent) to 7 (extremely severe). (c) Fagerström Test for Nicotine Dependence (FTND) Arabic version which has been validated for use among Arabic speaking population (28). In this study, nicotine dependence was classified into two categories; low dependence (LD; FTND < 4) and high dependence (HD; FTND ≥ 4). (d) Reasons for Quitting scale (RFQ) (29), which gathers information about motivations and reasons of smokers to quit smoking. The RFQ is composed of 20 self-report items and measures intrinsic and extrinsic motivation for quitting smoking. It consists of two intrinsic motivation subscales, self-control (e.g., “To show myself I can quit if I really want to”) and health concerns (e.g., “Because I'm concerned that smoking will shorten my life”), and two extrinsic motivation subscales, immediate reinforcement (e.g., “To save money that I spend on cigarettes”) and social pressure (e.g., “Because someone has given me an ultimatum to quit”).

The collected data was revised, coded, tabulated, and introduced to a PC using Statistical package for Social Science (SPSS 20). Data was presented, and suitable analysis was done according to the type of data obtained for each parameter.

- Descriptive statistics:
  1. Mean and Standard Deviation (±SD) for numerical data.
  2. Frequency and percentage for non-numerical data.
- Analytical statistics:
  1. Student *T*-Test was used to assess the statistical significance of the difference between two study group means.
  2. Chi-Square test was used to examine the relationship between two qualitative variables.
  3. Correlation analysis (using Pearson's and Spearman's methods): To assess the strength of association between two quantitative variables. The correlation coefficient denoted symbolically “r” for Pearson correlation and “rs” for Spearman correlation defines the strength (magnitude) and direction (positive or negative) of the linear relationship between two variables.
  4. Linear regression was used to test and estimate the dependence of a quantitative variable based on its relationship to one or more independent variables.

## Results

### Sample Description

The study included two groups; a group of patients with schizophrenia (*n* = 346; males 232, females 114) and a group of individuals with no mental illnesses (*n* = 150; males 93, females 57). The mean age of the group with schizophrenia was 33.28 years (±9.57 SD) and for the other group 36.97 years (±10.52 SD). The levels of illiteracy (13.9%) and unemployment (21.7%) were higher among patients with schizophrenia than in non-schizophrenia group (0.7 and 6.7%, respectively) (Table 1).

**Table 1:** Comparison between smokers with schizophrenia and smokers with no mental illness regarding socio-demographic data.

		**Smokers with no mental illness**		**Smokers with schizophrenia**		***t*****-test**	
		**Mean**	**SD**	**Mean**	**SD**	***p*****-value**	**Sig**.
Age		36.97	10.52	33.28	9.57	<0.001	S
		***N***	**%**	***N***	**%**	**Chi square test**	
						***p*****-value**	**Sig**.
Gender	Male	93	62.0	232	67.1	0.277	NS
	Female	57	38.0	114	32.9		
Marital	Unmarried	60	40.0	165	47.7	0.114	NS
	Married	90	60.0	181	52.3		
Education	Uneducated	1	0.7	48	13.9	<0.001	S
	Basic	10	6.7	64	18.5		
	High school	26	17.3	63	18.2		
	Diploma	56	37.3	119	34.4		
	College	57	38.0	52	15.0		
Job	Non functioning	10	6.7	75	21.7	<0.001	S
	Functioning	140	93.3	271	78.3		
Income	Not adequate	72	48.0	195	56.4	0.086	NS
	Adequate	78	52.0	151	43.6		

As regard patients' symptoms profile, they had higher positive scale scores (Mean ± SD = 25.01 ± 5.08) than negative scale scores (Mean ± SD = 18.64 ± 8.05).

### Pattern of Use and Severity of Tobacco Dependence Among Patients

Comparing the two groups, a statistically significant difference in the age of starting smoking was detected as the patients started at an earlier age than the other group (mean: patients with schizophrenia 22.15 ± 6.17 SD, non-schizophrenics 23.93 ± 5.89 SD, *p* = 0.003). However, patients with schizophrenia had statistically significant lower dependency scores using FTND (cases 6.6 ± 1.56, controls 7.12 ± 1.43, *p* = < 0.001) (Table 2).

**Table 2:** Comparison between smokers with schizophrenia and smokers with no mental illness regarding age, pattern of smoking and FTND score.

		**Smokers with no mental illness**		**Smokers with schizophrenia**		***t*****-test**	
		**Mean**	**SD**	**Mean**	**SD**	***p*****-value**	**Sig**.
Age of smoking		23.93	5.89	22.15	6.17	0.003	S
FTND		7.12	1.43	6.60	1.56	< 0.001	S
		***N***	**%**	**N**	**%**	**Chi Square**	
						***P*****-value**	**Sig**.
1st Cig	After 60 min	17	11.3	64	18.5	< 000.1	S
	30–60 min	52	34.7	52	15.0		
	6–30 min	59	39.3	95	27.5		
	Within 5 min	22	14.7	135	39.0		
No. of cig/d	< 10	3	2.0	78	22.5	< 0.001	S
	11–20	116	77.3	130	37.6		
	21–30	17	11.3	95	27.5		
	>30	14	9.3	43	12.4		

The number of cigarettes per day was significantly (*P* < 0.001) higher among patients group as 39.9% of patients with schizophrenia smoked more than 20 cigarettes per day compared to only 20.6% of the group without schizophrenia. Moreover, patients preferred their first cigarette within 5 min of awakening (39%) compared to the other group (14.7%) (*P* < 0.001).

On the other hand, there is a statistically significant negative correlation (*r* = −0.158) between positive scale score and age of starting smoking (the younger the age of start smoking, the higher the positive scale score), and a statistically significant positive correlation between positive scale score and FTND score (*r* = 0.246) (the higher the FTND score, the higher the positive scale score). This suggests that patients with more positive symptoms start smoking early and have more dependency score on FTND. However, no significant correlations were detected with negative scale and general scale scores (Table 3).

**Table 3:** Correlations between age of starting smoking, FTND score, RFQ scale, and PANSS.

***N*** **=** **346**		**Age of starting smoking**	**FTND-S**	**RFQ-INH**	**RFQ-INS**	**RFQ-EXI**	**RFQ-EXS**
Positive score	R	−0.158	0.296	0.483	0.245	−0.109	−0.123
	*p*-value	0.003	< 0.001	< 0.001	< 0.001	0.043	0.022
Negative score	R	0.068	0.038	0.070	−0.042	−0.102	0.002
	*p*-value	0.205	0.485	0.191	0.433	0.056	0.970
General	R	0.014	0.085	0.265	0.048	−0.109	0.028
	*p*-value	0.793	0.114	< 0.001	0.377	0.043	0.607

Upon performing linear regression analysis for predictors of FTND score, it was revealed that only age (0.041, 95% CI: 0.03–0.05), sex (−0.917, 95% CI: −1.18 to −0.65, ref. male), and schizophrenia (−0.398, 95% CI: −0.69 to −0.1) were significant predictors of FTND score (Table 4). Moreover, in the patients group, linear regression analysis for predictors of FTND score revealed that only age (0.043, 95% CI: 0.03–0.06), sex (−1.145, 95% CI: −1.45 to −0.84, ref. male), positive score (0.11, 95% CI: 0.08–0.14), and negative score (0.041, 95% CI: 0.02–0.06) were significant predictors of FTND score (Table 5).

**Table 4:** Linear regression analysis for predictors of FTND score in both groups.

	**Regression**	**95.0% confidence**	***p*-value**
	**coefficient**	**interval**	
Schizophrenia (Ref.: Non-schizophrenic)	−0.398	−0.69 to −0.1	0.008
Sex (Ref.: Male)	−0.917	−1.18 to −0.65	< 0.001
Age	0.041	0.03 to 0.05	< 0.001
Marital (Ref.: Unmarried)	−0.202	−0.49 to 0.08	0.163
Job (Ref.: Non-Functioning)	−0.341	−0.69 to 0.01	0.054
Basic (Ref.: uneducated)	0.223	−0.3 to 0.75	0.402
High school (Ref.: uneducated)	0.333	−0.17 to 0.84	0.196
Diploma (Ref.: uneducated)	0.320	−0.15 to 0.79	0.183
College (Ref.: uneducated)	0.441	−0.07 to 0.95	0.091

**Table 5:** Linear regression analysis for predictors of FTND in patients with Schizophrenia.

	**Regression**	**95.0% confidence**	***p*-value**
	**coefficient**	**interval**	
Gender (Ref.: Male)	−1.145	−1.45 to −0.84	< 0.001
Age	0.043	0.03 to 0.06	< 0.001
Positive score	0.111	0.08 to 0.14	< 0.001
Negative score	0.041	0.02 to 0.06	< 0.001
marital (Ref.: Unmarried)	−0.294	−0.62 to 0.04	0.082
job (Ref.: Non-Functioning)	−0.154	−0.51 to 0.2	0.395
Basic (Ref.: uneducated)	0.139	−0.38 to 0.65	0.597
High school (Ref.: uneducated)	0.455	−0.05 to 0.96	0.078
Diploma (Ref.: uneducated)	0.321	−0.15 to 0.79	0.178
College (Ref.: uneducated)	0.155	−0.39 to 0.7	0.574

Moving to specific symptoms, this study found statistically significant positive correlation, between number of cigarettes and severity of delusions, conceptual disorganization, hallucination, excitement, hostility, anxiety, preoccupation, and poor impulse control and statistically significant negative correlation with motor retardation and poor attention (Table 6).

**Table 6:** Correlation between symptoms of schizophrenia (PANSS) and the number of cigarettes and time spent before first cigarette.

	**Number of**		**Time before 1st**	
	**cigarettes**		**cigarette**	
	***N*** **=** **346**		***N*** **=** **346**	
	**rs**	***p***	**rs**	***p***
Delusions	0.147	0.006	−0.160	0.003
Conceptual disorganization	0.428	< 0.001	−0.417	< 0.001
Hallucinations	0.213	< 0.001	−0.201	< 0.001
Excitement	0.287	< 0.001	−0.286	< 0.001
Grandiosity	0.055	0.307	−0.085	0.115
Suspiciousness/ Persecution	0.085	0.115	−0.106	0.048
Hostility	0.185	0.001	−0.227	< 0.001
Blunt affect	0.018	0.740	−0.002	0.968
Emotional withdrawal	−0.010	0.852	0.042	0.434
Poor rapport	−0.029	0.591	0.049	0.361
Passive/apathetic social withdrawal	0.003	0.950	0.005	0.922
Difficulty in abstract thinking	0.035	0.513	0.011	0.837
Lack of spontaneity & flow of conversation	−0.019	0.726	0.025	0.643
Stereotyped thinking	0.042	0.434	−0.030	0.584
Somatic concern	0.079	0.143	−0.125	0.020
Anxiety	0.157	0.003	−0.138	0.010
Guilt feelings	0.013	0.816	−0.055	0.306
Tension	−0.051	0.343	0.053	0.326
Mannerism & posturing	0.099	0.067	−0.087	0.107
Depression	0.035	0.519	−0.020	0.704
Motor retardation	−0.178	0.001	0.165	0.002
Uncooperativeness	0.011	0.843	0.001	0.985
Unusual thought content	−0.035	0.518	0.031	0.563
Disorientation	0.045	0.408	−0.087	0.106
Poor attention	−0.138	0.010	0.157	0.003
Lack of judgment & insight	0.029	0.587	−0.008	0.883
Disturbance of volition	0.096	0.074	−0.076	0.157
Poor impulse control	0.226	< 0.001	−0.219	< 0.001
Preoccupation	0.150	0.005	−0.139	0.010
Active social avoidance	0.014	0.796	−0.043	0.422

Regarding correlation between symptomatology and time spent before the first cigarette, this study found statistically significant negative correlation between time spent before the first cigarette and severity of delusions, conceptual disorganization, hallucination, excitement, persecution, hostility, somatic concern, anxiety, preoccupation, and poor impulse control. While a statistically significant positive correlation was found with motor retardation (Table 6).

### Reasons for Quitting

Assessing the reasons for quitting, significant differences in intrinsic reasons were detected. This includes “health concerns” (mean: patients group 2.63 ± 1.02, non-schizophrenic group 2.17 ± 1.13) with a *P*-value of < 0.001 and “self-control” (mean: patients group 1.92 ± 1.06, non-schizophrenic group 1.69 ± 0.88) with a *P*-value of 0.017. Patients with schizophrenia were more concerned with health risk and to gain self-control over smoking than their counterparts from the other group. However, no statistically significant differences were detected as regard extrinsic factors; immediate reinforcement and social pressure as reasons to quit.

Moreover, regarding PANSS positive scale score, statistically significant positive correlation with health concern subscale and with self-control subscale were detected. On the other hand, negative correlations with immediate reinforcement subscale and social pressure subscale were also found. This suggests that patients with more positive symptoms are more concerned with intrinsic reasons (i.e., health and self-control) to quit smoking and less concerned with extrinsic reasons (i.e., less affected by immediate reinforcement and social pressure) (Table 3).

Furthermore, statistically significant positive correlation between general scale score and health concern subscale and negative correlation with immediate reinforcement subscale were detected. This implies that patients with schizophrenia who have higher scores on the general scale of PANSS are more concerned with health but less concerned with immediate reinforcement (Table 3).

## Discussion

Schizophrenia is associated with mortality rates that are two to three times higher than the general population. The number of deaths due to cardiovascular disease is high and could be reduced by lifestyle modification (2, 30). Kelly et al. (11) reported a significant risk of increased mortality from smoking in patients with schizophrenia which was mainly evident in the middle ages (35–54 y). This implies the importance of a better understanding of the smoking problem among patients with schizophrenia, and the inclusion of the assessment, and management of smoking in routine treatment strategies (9).

When assessing age of starting smoking, this study found that patients with schizophrenia start in a younger age, which may suggest that smoking might be involved in pathophysiology of schizophrenia or smoking may be an early sign of prodromal symptoms. A birth cohort study (31) links cigarette smoking to the prodromal phase of schizophrenia and suggested that impaired nicotinic neurotransmission is involved in the pathophysiology of schizophrenia. However, another study conducted by Wade et al. (32), reported that patients with schizophrenia began smoking around the same age as healthy controls, i.e., in their teenage years.

On the other hand, the current study found that patients with schizophrenia had lower dependency scores on FTND compared to the other group. A possible explanation for this is the high prices of cigarettes due to increased taxes especially in the recent years, which is a burden to the patients with schizophrenia who already suffer from financial problems. Another possible explanation is that most of patients with schizophrenia in Egypt live with their families who control their cigarettes supply. This result comes in concordance with several studies that indicated that nicotine dependence and the number of cigarettes per day are not higher and even sometimes similar to those without schizophrenia (33). However, other studies (3, 34, 35) found that people with schizophrenia are heavier smokers and had high levels of nicotine dependence scores.

The relation between smoking and positive and negative symptoms have always been debatable. Results of different studies have yielded various outcomes (4, 36, 37) with some reporting smoking related to positive symptomatology, to negative, to both positive, and negative symptomatology (38, 39) or to neither positive nor negative symptoms (37).

On assessing patients with positive symptoms, this study found that patients with more positive symptoms start smoking early and have more dependency score of FTND of cigarettes smoking. Yet, no significant correlations between negative symptoms or general symptoms severity and age of patients, age of start smoking, or cigarettes dependency were detected. This comes in concordance with work done by Ziedonis et al. (40) who found that heavy smokers (here defined as >25 cigarettes per day) had the most positive symptoms and a significantly lower number of negative symptoms. In addition, a cohort study done by Aguilar et al. (41) found that highly dependent smokers had the most severe positive symptoms of schizophrenia. Moreover, it was reported that tobacco use, and weight gain/obesity were associated with increased severity of symptoms of schizophrenia and decreased functioning (42).

However, upon performing linear regression analysis, both positive and negative scores were significant predictors of FTND score. This comes in concordance with study done by Patkar et al. (13) for assessments of nicotine dependence in relation to PANSS who found significant positive correlations between Fagerstrom scores and the total negative symptom score and scores on the negative symptom subscales of blunted affect, social withdrawal, difficulty in abstract thinking, and stereotyped while positive symptoms were not significantly associated with smoking.

On the other hand, morning smoking preference was positively correlated to conceptual disorganization and excitement in this sample. As regard the correlation with conceptual disorganization, this may be explained by the possibility that nicotine use enhances performance in several domains of cognitive functioning which may be reflected on process of thinking and goal-directed sequencing. Thus, morning smoking may be preferred because it might improve this thinking processing from the start of the day. The relaxing effect of nicotine may also explain the correlation between morning smoking and excitement (43). This study also linked the number of cigarettes and time spent before the first cigarette to certain symptoms including delusions, hallucinations, conceptual disorganization, excitement, hostility, anxiety, and poor impulse control.

When it comes to quitting, Evins et al. (44) observed that persons with schizophrenia are often highly motivated and persistent in their attempts to quit smoking despite having long histories of smoking. This study found that patients with schizophrenia are more concerned with intrinsic factors, i.e., heath risk and self-control as reasons to quit. These results are in concordance with a study done by Addington et al. (45) who found the chief reasons for quitting smoking in patients with schizophrenia could be health concerns, which ultimately reinforce these individuals to quit. However, this is not in concordance with other studies (46) which found that people with schizophrenia were significantly less likely to consider quitting for health concerns.

On correlating positive and negative symptoms with reasons for quitting, we found that patients with more positive symptoms are more concerned with intrinsic reasons (with health and self-control) and less concerned with extrinsic reason (less affected by immediate reinforcement and less affected by social pressure). However, no significant correlation between negative symptoms and the subscales of reasons for quitting was detected.

## Limitations

The main limitation of the study is its non-generalizability. It included a not well-representative community sample; as the sample was only collected from Institute of Psychiatry, Ain Shams University Hospitals.

## Conclusion

Tobacco is one of the major contributors to major health issues in patients with schizophrenia. It seems that there is some sort of a link between some positive symptoms of schizophrenia and severity of tobacco dependence, number of cigarettes, and morning smoking preference. It also appears that patients with schizophrenia who have more severe positive symptoms are more inclined to quit smoking; mainly for health concerns. Thus, it is important for psychiatrists to investigate these issues when assessing patients with schizophrenia especially their readiness to quit. Addressing tobacco use during the course of treatment might have positive outcomes on schizophrenia and health related issues and consequently lead to decreased morbidities and mortalities.

## Ethics Statement

This study was carried out in accordance with the recommendations of the ethical committee of Faculty of Medicine, Ain Shams University. The protocol was approved by the ethical committee of Faculty of Medicine, Ain Shams University. All subjects gave written informed consent.

## Author Contributions

NN, GT, ME, HE, and LA contributed to conception and design of the study. LA was the researcher responsible for the field work and organized the database. MY revised all the collected data and performed the statistical analysis. HE collated all results and wrote the first draft of the manuscript. All authors contributed to the revision, read and approved the submitted version.

### Conflict of Interest Statement

The authors declare that the research was conducted in the absence of any commercial or financial relationships that could be construed as a potential conflict of interest.
